# Morphology, Electrical and Optical Properties of Cu Nanostructures Embedded in AZO: A Comparison between Dry and Wet Methods

**DOI:** 10.3390/mi13020247

**Published:** 2022-02-01

**Authors:** Stefano Boscarino, Maria Censabella, Melanie Micali, Marco Russo, Antonio Terrasi, Maria Grazia Grimaldi, Francesco Ruffino

**Affiliations:** 1 Dipartimento di Fisica e Astronomia “Ettore Majorana”, Università di Catania, Via S. Sofia 64, 95123 Catania, Italy; maria.censabella@ct.infn.it (M.C.); melanie.micali@ct.infn.it (M.M.); marco.russo@ct.infn.it (M.R.); antonio.terrasi@ct.infn.it (A.T.); mariagrazia.grimaldi@ct.infn.it (M.G.G.); 2 CNR-IMM, Via S. Sofia 64, 95123 Catania, Italy

**Keywords:** copper, nanostructures, AZO, laser ablation, dewetting, solar cells

## Abstract

Herein, Cu nanostructures are obtained by solid-state dewetting of 9 nm copper layer (dry) or by ablating copper target, using a nanosecond pulsed laser at 1064 nm, in acetone and isopropyl alcohol (wet). The Cu nanostructures are embedded in aluminum-doped zinc oxide layer. Then, the electrical, optical, and morphological properties of the two kinds of systems, as a function of their synthesis parameters, are investigated. The aim is to compare the two fabrication methods and select the main conditions to achieve the best system for photovoltaic applications. The main differences, exhibited by the wet and dry processes, were in the shape and size of the Cu nanostructures. Dewetting in nitrogen produces faceted nanoparticles, with an average size below 150 nm, while laser ablation originates spherical and smaller nanoparticles, below 50 nm. Dry system underwent to thermal annealing, which improves the electrical properties, compared to the wet system, with a sheet resistance of 10^3^ vs. 10^6^ Ω/sq, respectively; finally, the dry system shows a maximum transmittance of 89.7% at 697 nm, compared to the wet system in acetone, 88.4% at 647 nm, as well as in isopropyl alcohol, 86.9% at 686 nm. Moreover, wet systems show higher transmittance in NUV.

## 1. Introduction

In the 21st century, the advent and development of nanoscience and nanotechnology has allowed for the widespread use of metal nanostructures in different fields, such as photonics, optoelectronics, biosensors, and photovoltaics [1,2,3,4,5]. The latter is an important and strategic research field, since solar cells can supply renewable and sustainable energy. Therefore, the task for researchers is to realize solar cell technology, of which the main characteristics are high efficiency, cost-effective, and environment-friendly in nature [6,7,8]. In terms of high efficiency, apart from the type of generation with their specific absorber layer and technology, the light harvesting performance of solar cell is a crucial factor, which heavily affects its efficiency [6,7,8,9,10,11,12]. In this context, metal nanostructures, thanks their unique physical, chemical, mechanical, and magnetic properties, connected to their small physical dimension and large surface/volume ratio, as well as the surface properties that dominate in nanostructures, with respect to bulk, and have gained increasing attention as one of the best solutions to ensure sunlight absorption enhancement [13]. For metallic nanostructures, the most known optical property is the surface plasmon resonance (SPR), which can be employed to improve the light harvesting performance of photovoltaic devices. The phenomenon of SPR takes place when the dimension of the particles becomes smaller or comparable with the wavelength of the incident light hitting them. In particular, SPR is a collective oscillation of free electrons, induced by an electromagnetic radiation and existing in a conduction band of metal, which becomes a localized surface plasmon resonance (LSPR), due to the reduction of the dimensions, which localize oscillations of electrons. The localized surface plasmon resonance occurs when the light hits the nanostructures and its frequency is resonant with the collective oscillations of electrons confined to the surface of the nanostructure, producing both strong absorption and scattering of incident light [9,12,13].

The intensity and wavelength of LSPR are influenced by many internal and external factors, such as the type of metal, particle size and shape, interparticles spacing, dielectric properties of the nanoparticles, and dielectric constant of the surrounding medium. Therefore, LSPR is expected to produce remarkable enhancement of the local electromagnetic field and light scattering, leading to an increase of the optical path length inside PV absorber material, which promotes an enhancement of the light harvesting performance in solar cells.

Until today, the most commonly studied and utilized materials are still noble metals, such as silver and gold, despite their costliness, which hinders the use and commercialization on large scale production; there are also various emerging alternatives, such as copper, aluminum, heavily doped semiconductors, metal oxides, and so on [1]. Among the alternatives, Cu is a great candidate because of its earth-abundant nature and low-cost, e.g., $0.009/g for Cu vs. $0.73/g for Ag and $55.7/g for Au [14,15,16], features that can support its use on large scale for the manufacturing of solar cells.

For solar cells application, the main characteristic of the selection of the metal is a strong surface plasmon at a desired resonance wavelength, which fits particularly in visible range (400–800 nm). The main reason gold and silver are the most interesting, studied, and developed materials in the field of nanostructures is due to their ability to support intense plasmon resonance that can be tuned throughout the UV–Vis–NIR range. In particular, the SPR wavelength for gold lies at about 550 nm while for silver lies in both visible and IR wavelength range (300–1200 nm) [17]. On the other hand, although copper was found to exhibit an LSPR in the range of 500–800 nm, based on Mie theory, due to the fact it undergoes to a rapid aerobic oxidization and gives the impression of a broad and weak LSPR at about 590 nm, copper has not attracted much attention, as compared to silver and gold [18]. Concerning to Cu nanostructures, the weak and broad peak is related to a large imaginary part, ranging from UV to NIR range, in their dielectric function, which, in turn, depends on interband transitions and intrinsic energy loss, due to electron collision. Between them, the main contribution is due to interbands transitions, which, as Mie theory predicted, showed an extended spectral overlap with the interband transitions, taking place to a considerable plasmon damping [19,20,21,22]. Nevertheless, a low-loss window, related to a significant drop in the imaginary part, from 620 to 720 nm, has been reported, which makes the latter one comparable to those of gold and silver and might allow for Cu nanostructures with intense SPR within that window.

Lately, many efforts have been made in order to obtain an intense and narrow peak as well as to prevent the oxidation of Cu towards CuO or Cu_2_O implementing various protection and synthesis methods. By means of Cu nanotriangle array, Chan G.H. et al. obtained an intense and narrow peak at 698 nm [23] while Sugawa et al. at 675 nm using Cu half-shell array [24] and lastly P. Zheng et al. realized Cu nanostructures with a cubic shape which produces an intense, narrow, and asymmetric LSPR peak at 585 nm [14]. Whereas, in order to avoid Cu oxidation, various approaches are employed, such as the preparation of Cu nanostructures in nonaqueous solvents, such as that in which P.S. Liù et al. worked on ethanol, glycol, and acetone [25], as well as R. Rawat et al., which worked on ethylene glycol and ethanol [26] or embedding the nanostructures into a different matrix [27]. In that last context, in the present work aluminum-doped zinc oxide (AZO) has been chosen as the embedding matrix for the Cu nanostructure because it is compatible with solar cells manufacturing and, more importantly, as an indium-free alternative transparent electrodes or indium-free buffer layer [28].

The main goal in the production of Cu nanostructure is to control the shape, morphology, and dimension because these features, with its chemical surrounding influencing the characteristics of the LSPR peak. Cu nanostructures can be produced by many known methods, which include chemical and physical methods, with their advantages and disadvantages being solid-state dewetting, nanosphere lithography, plasma synthesis, e-beam lithography, focused ion beam machining, chemical reduction, electrochemical synthesis, micro-emulsions, and laser ablation of solid target in liquid medium (LAL) [5,29,30]. Among “dry physical methods”, solid-state dewetting (SSD) offers cost-effective, time-efficient solutions and is fully compatible with the industrial manufacturing lines of solar cells, especially for thin film [31,32]. On the other hand, it requires sophisticated equipment and technology to deposit the thin film material and performs a thermal annealing under high controlled atmosphere. The SSD method is based on a deposition of a thin metal film, followed by a thermal annealing at temperatures well below the melting point of the metal. The annealing breaks up the metal film into nanostructures, on the substrate, which morphology depends, in the first instance, on the temperature of process and the interaction between the film and the substrate. 

While “wet physical methods”-like laser ablation of solid target in liquid medium (LAL) offers advantages, such as the simplicity of operation, environmentally-friendly technique, operating under ambient conditions in water or organic liquids, and a limited equipment requirement. The main expensive parts of the LAL setup are the laser system and target material employed.

Briefly, in a LAL process, the laser beam is focused (by an optical system) on the surface of a solid target and immersed in a liquid environment, which, absorbing the laser radiation, leads to the production of an expanding plasma plume, which contains the ablated material. The expansion of the plasma plume inside causes its cooling and shrinking and, at the same time, a reduction of the pressure and temperature. That condition is suitable for the nucleation and growth of the particles into the plasma plume. Lastly, when the plasma plume collapses, the particles are released into the surrounding liquid, forming a nanoparticle colloidal solution. In this technique, the key elements to obtain nanostructures with well-defined size and shape are the laser parameters (laser wavelength, pulse duration, laser fluence, and repetition rate) and liquid environment, where the ablation was carried out.

In conclusion, Cu nanostructures are obtained by solid-state dewetting of 9 nm Cu layer (dry) and ablating the Cu target using a nanosecond pulsed laser in acetone and isopropyl alcohol (wet). First of all, the Cu nanostructures morphology (shape and size) are investigated as a function of the involved method of synthesis and key parameters. In the SSD process, the morphology was studied as a function of the annealing temperature, ranging from 300 to 500 °C, while the laser ablation of a solid target in a liquid medium was studied as a function of the used liquid environment. Once the Cu nanostructures are realized, they are embedded in aluminum-doped zinc oxide layer and investigated for the electrical, optical, and morphology properties of the whole system, as a function of AZO thickness. The aim is to compare the two methods and find the main conditions to achieve the best system for photovoltaic applications, especially from an optical point of view. The main differences, exhibited by wet and dry processes, were in the shape and size of the Cu nanostructures. Dewetting in nitrogen produces faceted nanoparticles, with an average size below 150 nm, while laser ablation originates spherical and smaller nanoparticles, below 50 nm. The dry system undergoes thermal annealing and improves the electrical properties, compared to the wet system, with a sheet resistance of 10^3^ vs. 10^6^ Ω/sq, respectively. Finally, the absorbance, transmittance, and reflectance spectra of systems, in the wavelength range of 250–1100 nm, were investigated. The dry system showed a maximum transmittance of 89.7% at 697 nm, compared to wet systems in acetone, 88.4% at 647 nm, and IPA, 86.9% at 686 nm. Moreover, wet systems showed higher transmittance in the NUV range.

We propose the present work as an opening perspective for more extended and technologically-related analysis. In fact, regarding this point, our related perspectives are:

I. The incorporation of the Cu nanostructures, as well, in other new-generation transparent conductive oxide (as Zr-doped indium oxide), as to carry out a comparison of the final device performances and choose the best transparent conductive oxide;
II. The incorporation of Al nanostructures, alternative to Cu nanostructures, in transparent conductive oxides, as to carry out a comparison of the final device performances and choose the best low-cost plasmonic nanoparticles;
III. The fabrication of prototypes of Si-based solar cell devices by growing Si-based active layers on the transparent conductive oxides/nanostructures systems, as well as testing the resulting prototypes performances.

Hence, the present work can be considered a first step towards the optimized nanostructures and transparent conductive oxides, in order to select the best combination for improved Si-based solar cell devices.

## 2. Materials and Methods

Aluminum-doped zinc oxide films were deposited on Corning glass substrate by RF magnetron sputtering, with a power density of 2.16 W/cm^2^, 7 cm target-substrate distance, Ar atmosphere, room temperature, and working pressure of 5.8 10^−3^ mbar. A ceramic AZO target, 2 wt.% Al_2_O_3_, was employed as a source material. Two sets of samples were produced differing for the AZO film thicknesses, 60 and 100 nm, respectively. In addition, a set of samples was fabricated by dewetting Cu film sputter-deposited on the AZO film. In this case, in particular, a 9 nm thick Cu film was deposited onto AZO Layer by sputtering using the Emitech K550X sputter coater, at a condition of 50 mA, 2 min. Thermal solid dewetting of Cu films was performed by a tubular oven by Carbolite Gero in saturated N_2_ atmosphere, in order to prevent spontaneous oxidation, with a temperature ranging from 300 to 500 °C, 1 h. Rutherford backscattering spectrometry (RBS) was employed to quantify the doses “A_element_” of the chemical elements Zn and Cu in the AZO/Cu/AZO system. Using the atomic density “d” of the materials, for AZO (7.22 × 10^22^ at./cm^3^) and Cu (8.48 × 10^22^ at./cm^3^), we determinated the thickness “t” of each layer as t= A_element_/d. A 2.0 MeV He^+^ ion beam at normal incidence, with the backscattered ions detected at an angle of 165°, was used. SimNra software [33] was employed to simulate the RBS spectra for the quantitative analysis. 

In addition, Cu nanostructures, in acetone and isopropyl alcohol have been synthetized by pulsed laser ablation from a metal target made by Cu (thickness of 1.0 mm, purity of 99.99%). In particular, a beam of Nd: yttrium aluminum Garnet YAG Laser (quanta-ray pro-series pulsed Nd: YAG laser (Spectra Physics, Santa Clara, CA, USA), 10 ns pulse, λ = 1064 nm, fluence 5 J/cm^2^, frequency of 10 Hz, 8 min, laser spot 2 mm was focalized by means of a 20 cm focal length lens on the target, at the bottom of a teflon vessel, filled with 8 mL of solvent. From the colloidal solutions, the Cu nanostructures were transferred on the surface of the AZO layer by the drop-casting method.

The AZO film surface and Cu nanostructures morphology was investigated by SEM, a field-emission Gemini 152 Carl Zeiss Supra 25. The analyses were performed by using the in-lens detector, with an aperture size of 30 μm, acceleration voltage of 3 kV, and a working distance of 3 mm. The four-point collinear probe method, by employing a Keithley 4200 semiconductor characterization system at room temperature, was used to measure the sheet resistance of system, R_sh_. Absorbance (Abs), transmittance (T), and reflectance ^®^ spectra of systems, in the wavelength range of 250–1100 nm, were obtained by using a Varian Cary 500 double beam scanning UV–VIS–NIR spectrophotometer. Moreover, the reflectance spectra have been measured in specular geometry at 20° using a calibrated sample as reference, while the direct transmittance were normalized to a 100% baseline, achieved by mounting the sample holder empty.

### 2.1. Image Analysis Processing and Procedure

Towards the determination of the size Cu nanostructures, in order to identify the nanoparticles and determine their size, a technique, known in the field of digital image processing, called the Hough transform, was used [34]. 

This technique assigns a predetermined geometric shape, here circles, and searches, in the space of its free parameters (in this case the coordinates of the circle and the radius), all the shapes to which at least one point of the image belongs. In the end, the shapes that interpolate the greatest number of pixels are the ones sought.

The method in question can only be successful if the image resolution is high enough, in other words each nanostructure must be represented by a reasonable number of points, otherwise the algorithm fails.

For the nanostructures examined, it was, therefore, decided to proceed as follows:

(1) Apply an edge detection filter: Sobel (raw Cu nanostructures) or Canny (AZO-covered Cu nanostructures);
(2) Convert the image obtained from the previous step into a sequence of two-dimensional coordinates;
(3) Apply the Hough transform; and
(4) Merge the circles very close to each other.

Even if this procedure could be highly time-consuming, from a computational point of view, a suitable choice of the starting parameters (mainly: a), the lowest distance between the circle and a point, for which the point is considered to hold to the circle, with (b) smaller and larger value for the circle, effectively reducing the computational time and getting reasonable results.

### 2.2. Dry and Wet Systems Preparation

**Figure 1:**
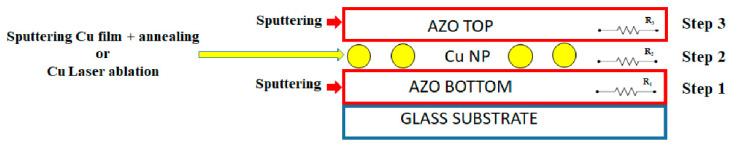
A scheme of the steps, with the involved processes, taking place to produce the dry and wet systems. Each layer is characterized by a sheet resistance, named R_1_, R_2_, and R_3_.

The systems, both dry and wet, were realized by three subsequent steps, Figure 1. 

The first step was the deposition of AZO bottom on Corning glass substrate by RF magnetron sputtering; then, the deposition of Cu nanostructure, on AZO bottom, and, finally, the deposition of AZO top to cover and protect the Cu nanostructures.

The second step, as a function of the involved method to produce Cu nanostructures, was different. In particular, in the case of thermal dewetting after the deposition of Cu thin film on AZO bottom, the Cu nanostructures were obtained by a thermal annealing of the glass substrate/AZO bottom/Cu stack. It leads to an energetically favorite set of Cu nanoparticles, at temperatures below the melting point. Lastly, the previous structure, with the formed Cu nanostructures, was covered with an AZO top layer. While, in the case of laser ablation process, a fraction of 100 µL, after a dispersion using ultrasonic processes, was collected from Cu nanostructures colloidal solution and poured on AZO bottom layer. The solvent has been evaporated, by means of a hot plate, at 95 °C, 45 min, with the structure glass substrate/AZO bottom/Cu nanostructure + solvent involved in that weak annealing. After the evaporation of the solvent, the AZO top layer was deposited by RF magnetron sputtering. In all systems, the AZO bottom and top layers had the same thickness.

## 3. Results and Discussion

### 3.1. Morphology Analysis

**Figure 2:**
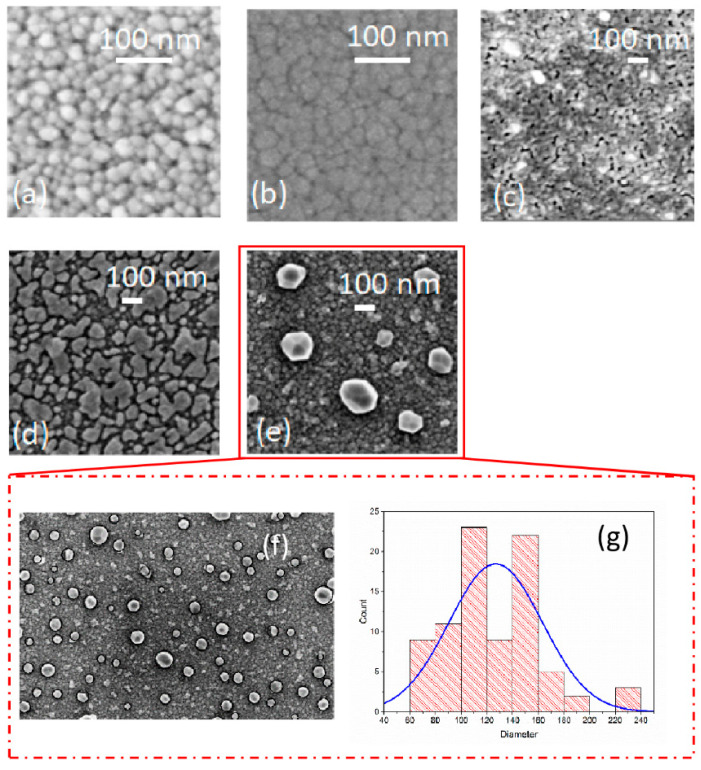
SEM images of (**a**) as deposited AZO botttom layer surface; (**b**) the surface of as deposited Cu on AZO bottom layer; (**c**) glass substrate/AZO bottom/Cu stack after a thermal annealing at 300 °C; (**d**) glass substrate/AZO bottom/Cu stack after a thermal annealing at 400 °C; (**e**) glass substrate/AZO bottom/Cu stack after a thermal annealing at 500 °C; (**f**) SEM image at low magnification (of image (**e**)), with fitting circles, obtained from our image analysis procedure; (**g**) particles size distributions.

Morphology and feature size of the Cu nanostructures, Figure 2, created on AZO bottom layer, as a function of the annealing temperature, were investigated using SEM. Figure 2a, shows the surface of AZO bottom layer and, as expected for this kind of deposition, we observed a symmetric granular structure, related to a highly (002) textured columnar polycrystalline films with a wurtzite structure [28]. The Cu material was deposited uniformly and conformal to the AZO bottom layer, Figure 2b, with an early stage of growth of thin film highlighted by the nucleation or phenomena of aggregation, as well as the AZO granular structure, glimpsed under the copper. After a thermal annealing of 300 °C, Figure 2c, we observed an early-stage dewetting with the creation of hole and agglomeration clearer. A 400 °C the solid-state dewetting process, Figure 2d, was achieved, and Cu nanostructures of different shape (irregular, spherical, worm-like, etc.) with wide dimensions were created. Increasing the temperature up to 500 °C, Figure 2e, we promoted more agglomeration with the formation of bigger Cu nanoparticles, faceted nanoparticles with an average size below 200 nm were produced. In particular, the size distribution of the latter sample, obtained by means of our analysis procedure, previously mentioned, consisting of particles, captured in a large portion of the sample, Figure 2e, with a size varying from 73 nm to 233 nm, Figure 2f.

The average size of particles is 123 ± 36 nm, Figure 2g. Therefore, SSD process will result into a particles distribution dominated by large sized faceted nanoparticles. As expected after the deposition of AZO top layer, SEM image, not reported here, showed our nanoparticles covered by a compliant and continuous AZO layer with an average size of 153 ± 51 nm. 

**Figure 3:**
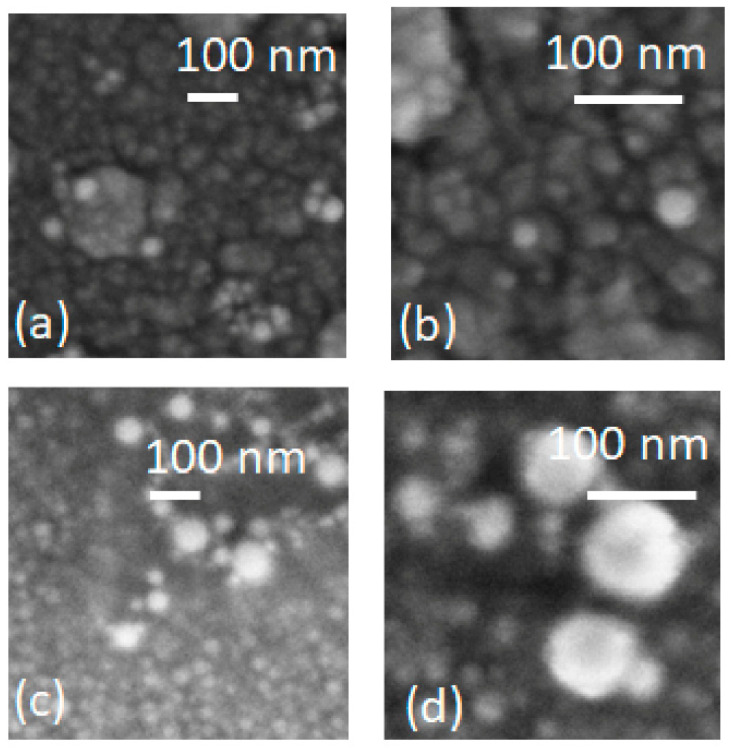
SEM images, at two different magnifications of Cu nanoparticles produced in acetone (**a**,**b**) and in IPA (**c**,**d**) depositated on AZO bottom layer.

Figure 3 reports SEM images of Cu nanostructures obtained by laser ablation in organic solvents at power density of 5 J cm^−2^ per pulse and for 8 min; then, they were deposited on AZO bottom layer. Specifically, Figure 3a,b reports on Cu laser ablation in acetone, while Figure 3c,d shows Cu laser ablation in IPA images, which were acquired at two magnifications, respectively. As we can see, laser ablation, both in acetone and IPA, produced nearly spherical nanoparticles, with an average diameter larger in IPA than in acetone. In particular, the size distribution consisted of nanoparticles, captured in a large portion of the sample, with a size varying from 22 to 52 nm in acetone and from 17 to 118 nm in IPA. The average size of particles in acetone was 33 ± 7 nm, while in IPA was 44 ± 22 nm. These results, in terms of average size and distribution, are in agreement with the behavior, as a function of dipole moment. The higher moment of acetone, prohibiting the growth mechanism, produces smaller nanoparticles [35,36].

Therefore, the LAL process will result in particle distribution dominated by small-sized, spherical nanoparticles. Moreover, laser ablation in acetone, compared with IPA, exhibit superior dispersivity and less aggregation, apart from the small average size of the spherical nanoparticles. In conclusion, once the Cu nanostructures are realized by wet and dry processes, the main differences exhibited were in the shape and size of the Cu nanostructures. Dewetting in nitrogen produces faceted nanoparticles, with a dimension below 240 nm, while laser ablation originates spherical and smaller nanoparticles, with a dimension below 50 nm.

### 3.2. UV–Vis–NIR Optical Analysis

**Figure 4:**
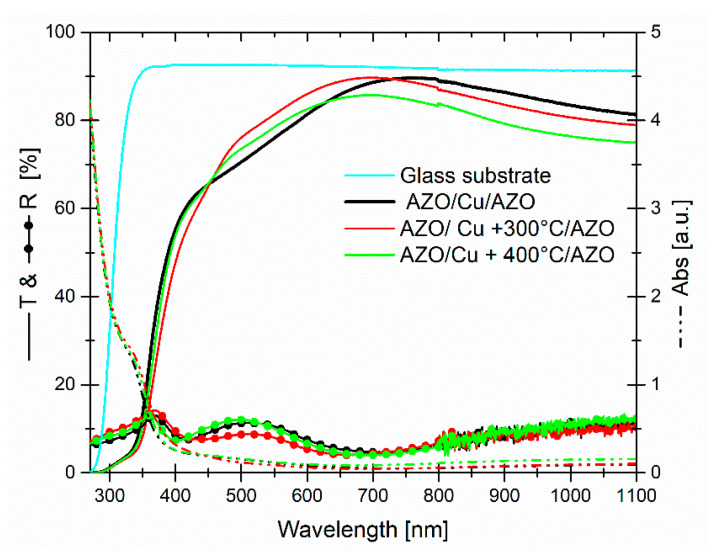
Transmittance, reflectance, and absorbance curves of dry systems, as a function of the annealing temperature. The transmittance of bare glass substrate is reported as the reference point.

Figure 4 reports the optical transmittance, reflectance, and absorbance of the dry systems, with an AZO thickness set at 100 nm. Moreover, the transmittance of the glass substrate is also reported, and the optical data includes the substrate contribution. 

As far as the transmittance is concerned, all systems showed a threshold around to 375 nm and, simultaneously, the absorbance in this UV region (from 375 to lower wavelength) increased considerably. While, in the visible-near infrared range (400–1100 nm), it was very low and decreasing mainly towards NIR range. The maximum of the transmittance, as a function of the annealing temperature, then the evolution of Copper structures embedded in AZO, was observed a blue-shift and at the same time a decreasing in the value of the maximum. Specifically: 89.6% at 759 nm, 89.7% at 701 nm, and 85.7% at 691 nm for AZO/Cu/AZO, AZO/Cu + 300 °C/AZO, and AZO/Cu + 400 °C/AZO, respectively. Among them the best performance, in terms of transmittance, the average value measured in the visible (400–800 nm) and VIS–NIR ranges (400–1100 nm) belong to AZO/Cu + 300 °C/AZO, with values of 80.7% and 81.4%, respectively. Reflectance curves, on the other hand, showed a complementary behavior, with respect to transmittance exhibiting a maximum, where the transmittance was low and vice versa. Anyway, for most of the wavelength range, the reflectance values for all systems were below 15%.

**Figure 5:**
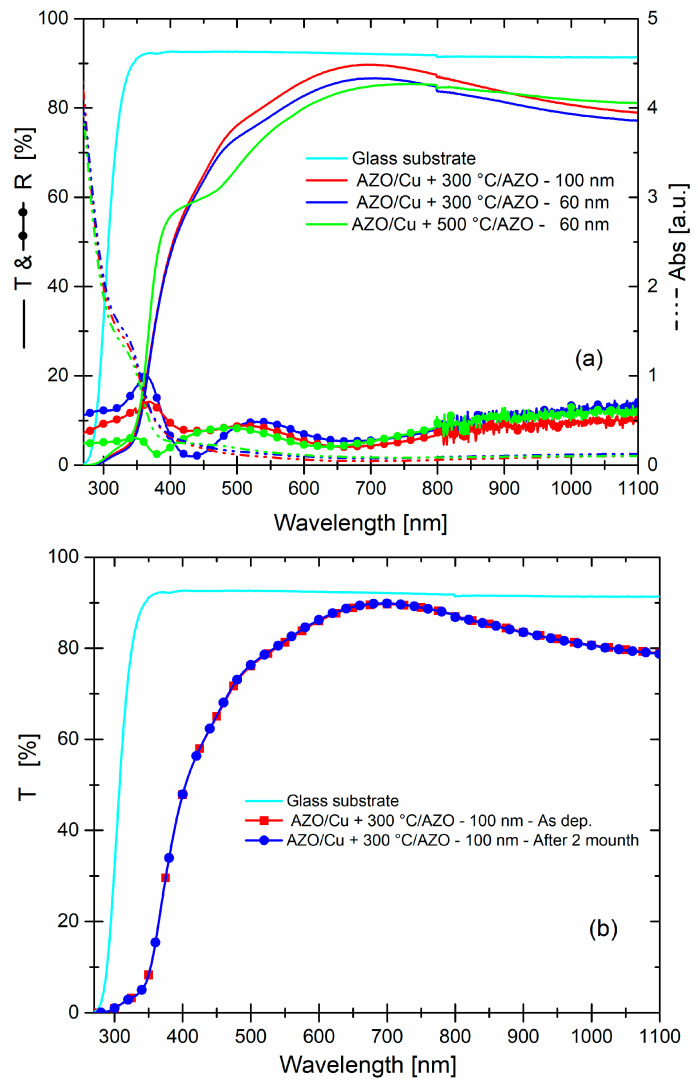
(**a**) Transmittance, reflectance, and absorbance curves of dry systems, as a function of the AZO thickness and annealing temperature. The transmittance of bare glass substrate is reported as the reference point. (**b**) It is reported the comparison between the transmittance of AZO_100 nm/Cu + 300 °C/AZO_100 nm was made after two months.

If AZO thickness was decreased to about 60 nm in the best AZO/Cu + 300 °C/AZO system, we observed (Figure 5a) the same behavior of the transmittance curve with 100 nm AZO but with decreased values, from 400 to 1100 nm. As we can see, this reduction can be related to an increased reflectance value, since absorbance values were equal. Moreover, if we add a thermal annealing at 500 °C, according to synthesis process, the transmittance values became worst, respectively, with values of 75.7% (400–800 nm) and 78.7% (400–1100 nm), although reflectance and absorbance values were low. 

That situation may be due to an increasing diffused part of the light, connected to the dimension of nanostructured Cu embedded in AZO. The stability of the optical properties of system has been observed as a function of the time, precisely until to two months after the synthesis, and is shown only for the transmittance that was unchanged, Figure 5b. 

**Figure 6:**
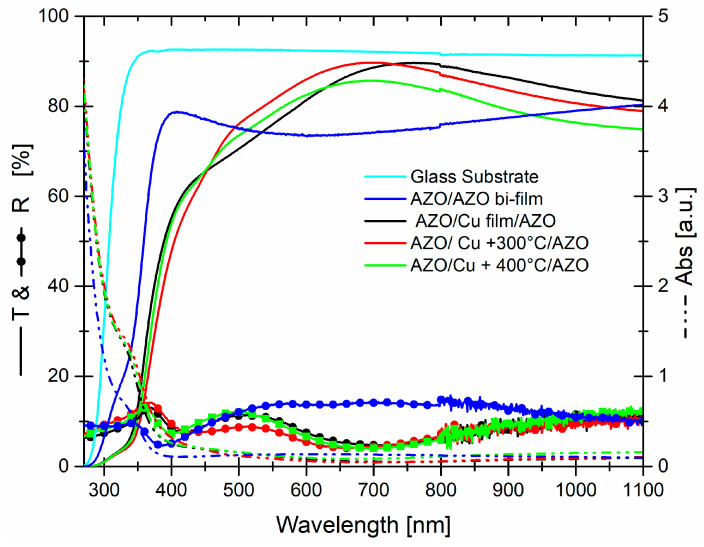
Transmittance, reflectance, and absorbance curves of dry systems, as a function of the annealing temperature. The transmittance of bare glass substrate, as well as the transmittance, reflectance, and absorbance of AZO bi film are reported as reference points.

A comparison of dry systems with a bi-film of two 100 nm AZO, the same thickness of AZO layers in the systems, Figure 6, highlighted some important points:

(1) The threshold was due, principally, to the direct optical band-gap of AZO layers. Photons with an energy higher than the optical band gap of AZO are absorbed by electrons with their promotion from valence band to conduction band. When Cu nanostructures are embedded in AZO, the presence of copper modified the threshold position, moving towards higher wavelengths and, at the same time, created a drop in the transmittance between 350–450 nm. Obviously, these modifications are drawbacks for the dry system, since they cut a portion of the transmitted light in this region and, at the same time, the other part was reduced.

(2) An overlook of absorbance, transmittance, and reflectance curves showed that the increasing in the transmittance of the dry systems, compared to 200 nm AZO layer in a wide range from 450 nm to 1100 nm, was clearly connected to a variation of reflectance. The Cu nanostructures embedded in AZO contributed to the increasing transmittance, at the expense of reduced reflectance.

**Figure 7:**
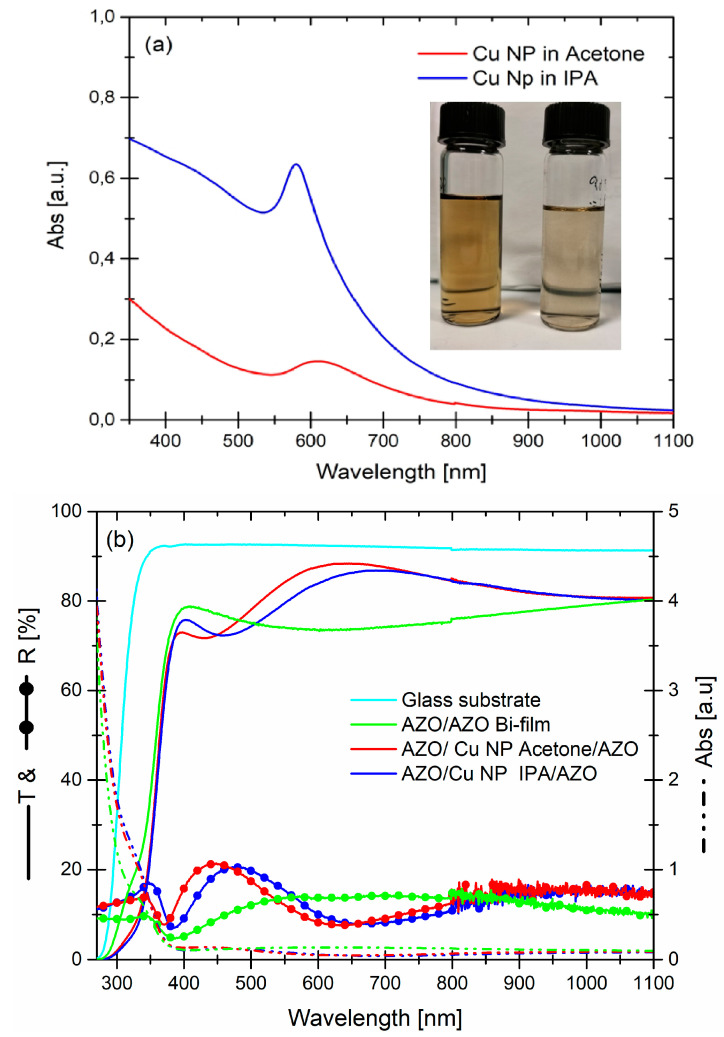
(**a**) Absorbance spectra of as-prepared Cu nanostructures in acetone (on left side of the photo) and IPA (on the right side of the photo), under 5 J cm^−2^ per pulse laser power density for 8 min. (**b**) Transmittance, reflectance, and absorbance curves of wet systems, as a function of the used solvent to produce the Cu nanoparticles. The transmittance of bare glass substrate, as well as the transmittance, reflectance, and absorbance of AZO/AZO bi-film, are reported as reference points.

Figure 7a showed an absorbance spectra of colloidal copper solution, prepared in acetone and IPA, at a laser ablation of 5 J cm^−2^, as well as at pulse for 8 min. Remember that our goal was to produce metal Cu nanoparticles. For this purpose, water (as a medium) was avoided because of it very high oxidation power during the production of nanoparticles in water [25], while the use of acetone and IPA is related to their characteristic of green and low-cost materials, with a low oxidation power because of their low/medium hygroscopicity [37].

The spectra exhibited a distinct SPR band and, along with a copper interband transition (IBT), dominated the absorbance in the region of short wavelength region. The position of the SPR band peaks, as a function of the solvent, was positioned at different wavelength, specifically at 610 nm for acetone and 580 nm for IPA. 

In terms of the whole spectra, the achieved Cu colloidal solution in acetone showed a red-shift of the SPR band, compared to IPA, along with difference in intensity and shape: the peak in acetone was weak and broad, while in IPA was intense and narrow. In the first instance, concerning the difference in intensity and peak position, we believed that the higher intensity arose from the increase amount of copper metal nanoparticles in the solution. However, a check of the target’s copper mass before and after the laser ablation process, in each solvent, revealed more Cu dissolved in acetone, 25 × 10^−5^ g, compared to IPA, 21 × 10^−5^ g. Therefore, these differences arise, principally, from the difference in size, larger in IPA than in acetone, with respect to the organic solvents. With their properties and the encapsulation of nanoparticles by carbon produced during laser ablation. 

Once the Cu colloidal solutions are produced, the nanoparticles were embedded in 200 nm AZO layer, according to the synthesis process, as previously mentioned. The optical transmittance, reflectance, and absorbance spectra of wet systems, as well as the 200 nm AZO layer and transmittance of the glass substrate, are reported in Figure 7b. Once again, the optical data included the glass substrate contribution.

As far as the transmittance is concerned, all systems showed, once again, a threshold around to 375 nm. Simultaneously, the absorbance in this UV region increased considerably, while in visible-near infrared range, it was very low and decreased towards a higher wavelength range. For the maximum of the transmittance, as a function of Cu nanoparticles (and used solvent) embedded in AZO, values of 88.4% at 647 nm for acetone and of 86.9% at 686 nm for IPA were observed.

Between them, the best performance, in terms of transmittance average value measured in the visible range (400–800 nm) and VIS–NIR range (400–1100 nm), belong to AZO/Cu NP in acetone/AZO with values of 82.8% and 82.4%, respectively. Moreover, in this case the threshold is almost similar between AZO bi-film and wet systems. 

Reflectance curves, on the other hand, showed a complementary behavior respect to transmittance exhibiting a maximum where the transmittance was low and viceversa. Anyway, the reflectance values for all Systems were below 20%. Also in this case, we observed that the increasing in transmittance of the wet system, from 450 nm to higher wavelength, was related to a decreasing in reflectance e absorption, compared to bare AZO. 

**Figure 8:**
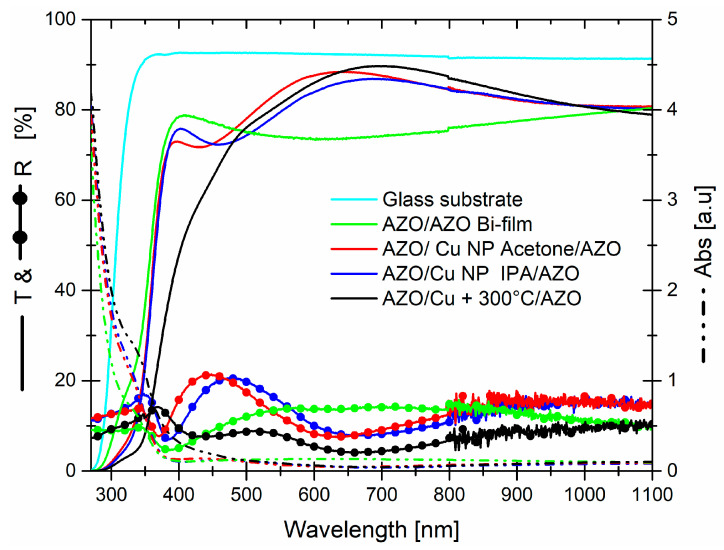
Comparison of transmittance, reflectance, and absorbance among AZO/Cu + 300 °C/AZO dry system, AZO/Cu in acetone /AZO and AZO/Cu in IPA/AZO wet systems. The transmittance of bare glass substrate, as well as the transmittance, reflectance, and absorbance of AZO/AZO bi-film, are reported as reference points.

In conclusion, we compared (Figure 8) the whole optical properties of wet and dry systems, along with the transmittance of the glass substrate and 200 nm AZO layer, which are reference points. The first difference come to light was the position of threshold, in wet systems the threshold was the same of AZO bi-layer, while in dry system it was positioned towards higher wavelength, that last condition wasn’t the best one because of narrowing of light transmission window. 

Moreover, in the wavelength range around the threshold, 350–450 nm, wet system allowed a light transmittance comparable to AZO bi-film and higher than dry system. If we observed the absorbance and reflectance curves carefully, although the dry system reflected less; compared to the wet system, the dry system was more absorbent with an absorption, which started earlier than it produced a reduction in the transmittance. We supposed that, this difference can be ascribed to the different method of covering the bottom AZO film. 

Although the maximum transmittance value of the dry system was slightly higher than the wet system’s, the wider transmission window, with the best performances in terms of transmittance average value measured in the VIS–NIR range, and the best position of the maximum, well-positioned, with respect to the solar spectrum, led a tendency in favor of the wet system (for a choice between the two), especially for AZO/Cu NP acetone/AZO. The differences in the reflectance curves, with the same absorption from 450 nm, might be attributed to a major scattering of light. Moreover, it is well-known that a material with a higher refractive index, deposited on metal, gives a better antireflective effect. Concluding this part, several model and simulation, based on the thickness, refractive index and extinction coefficients of the materials involved in these structures, i.e., TCO/metal/TCO, have been proposed to describe the optical properties [38,39,40]. Here, we are motivated to achieve a maximum transmittance over a broad spectrum, from NUV to NIR; therefore, our strategy was to maximize the transmittance varying the TCO thickness (our best condition was at 100 nm) and morphology of metal thin film/nanoparticles. Observing their optical properties, that systems are suitable for to be placed on the front surface of solar cell, but further studies are in progress in order to investigate and understand the scattered light, both transmitted and reflected, for a possible tuning of their optical properties in order to be suitable as back reflector.

### 3.3. Electrical Properties

The electrical properties of the wet and dry systems, along with AZO or Cu films, both as dep and after a process, were characterized by sheet resistance (R_sh_) measurement at room temperature, as described in Table 1. 

Starting from the bottom AZO films layer, deposited at room temperature, we can see it showed a very high sheet resistance depends on very thin thickness and even more on the nature of TCO and deposition process parameters (e.g., room temperature deposition). As expected, after a thermal treatment, that improves the crystalline quality, doping of Al and strain [28,38], the sheet resistance of the bottom AZO film improves up to three orders of magnitude, and a higher supply temperature results in greater improvements. Beyond 400 °C no significant improvement was observed. 

When Cu is deposited on as dep AZO bottom, the sheet resistance is still the same because of a non-continuous film Cu made of Cu islands with various sizes, randomly and uniformly distributed on whole bottom AZO, see Figure 1 That stack, both at two films and three films configuration, after a thermal treatment, necessary to achieve the Cu nanostructures, improved its sheet resistance. The R_sh_ value of the stack can be expressed considering the bottom AZO and Cu layers as two resistors connected in parallel where the sheet resistance of the whole stack is controlled by bottom AZO film; the Cu coalesced gradually towards the non-continuous film of bigger nanoparticles and bottom AZO improved its Rsh, as a function of temperature. Finally, with the room temperature deposition of AZO top layer, the system, bottom AZO + Cu nanostructures + Top AZO, can be seen as three resistors connected in parallel, see Figure 1:

1/R_system_ = 1/R_1_ + 1/R_2_ + 1/R_3_, (1)

where the whole R_sh_ is controlled by AZO bottom layer (R_1_) because of the lower sheet resistance compared to the not annealed AZO top layer and the non-continuous Cu. On the other side, wet systems suffered of poor sheet resistance because of AZO films and Cu nanoparticles are deposited at room temperature and they do not undergo at any thermal treatment, the thermal treatment in hot plate (95 °C, 45 min) is irrelevant. In order to overcome the sheet resistance value of wet systems a preliminary work, integrating wet and dry processes, by adding a thermal treatment of AZO bottom layer in the first step of wet procedure, highlighted that is possible to realize a new system with the same optical properties of the best wet system with a sheet resistance three order of magnitude lower. Of course, this route will be followed-up, in order to improve further the electrical properties keeping the optimum optical properties.

## 4. Conclusions and Perspectives

Although a lot of study and scientific research have been done in this fields, mainly working with gold and silver, to the best our knowledge, it still is a great challenge to improve and integrate Cu nanostructures in solar cells, in order to enhance the solar cell efficiency over the whole visible and NIR spectrum. The aim of this research is to enrich the knowledge of Cu nanostructures synthesis and provide a system that is easily integrated with solar cells, such that, with its electro-optical properties, it can support an improvement respect to state of the art. 

In conclusion:

- Concerning the image analysis processing, used here to look for particles and their dimension, the size distribution results, even if preliminary, are fully reasonable, in our opinion. As perspective, we are trying to improve the size calculation algorithm by an auto-tuning process of all the involved parameters. Further, for particles with irregular shape and wide dimension, e.g., annealing at 400 °C for Cu, we are studying the use of machine learning to develop a novel clustering technique that customizes, for our purposes, the ELBG algorithm [41].
- The dry system underwent thermal annealing to improve the electrical properties, compared to the wet system, with a sheet resistance of 10^3^ vs. 10^6^ Ω/sq, respectively. The transmittance spectra of systems, in the wavelength range of 250–1100 nm, showed the best dry system at a maximum transmittance of 89.7% at 697 nm, compared to the best wet systems in acetone, 88.4% at 647 nm, as well as in IPA, 86.9% at 686 nm. Although the maximum value of the dry system was slightly higher than wet systems, the higher transmittance in NUV range, wider transmission window (with the best performance, in terms of transmittance average value, measured in the VIS–NIR range), and best position of maximum (well-positioned, with respect to the solar spectrum) endorsed the AZO/Cu NP acetone/AZO wet system.

We believe that these results, with low-cost and simple-fabricated methods, supply practical data for a better utilization of cost-effective Cu nanostructures, in order to improve the light harvesting performance of photovoltaic devices.

## Figures and Tables

**Table 1:** Sheet resistances of: 100 nm AZO layers as dep and after a thermal treatment in the range of 300–500 °C; AZO/Cu stack as dep and and after a thermal treatment in the range of 300–500 °C; AZO/Cu + thermal annealing/AZO for dry systems AZO/Cu + solvent /AZO for wet systems.

AZO Layer	Sheet Resistance [Ω/Sq]
AZO bottom	2.2 • 10^6^
AZO top	4.3 • 10^6^
AZO + 300 °C	1.3 • 10^4^
AZO + 400 °C	2.1 • 10^3^
AZO + 500 °C	1.5 • 10^3^
Dry samples	Sheet resistance [Ω/Sq]
AZO bottom/ Cu	8.5 • 10^6^
AZO bottom/Cu + 300 °C	1.4 • 10^4^
AZO bottom/Cu + 400 °C	4.5 • 10^3^
AZO bottom/Cu + 500 °C	2.3 • 10^3^
AZO bottom/Cu +300 °C/AZO top	7.4 • 10^4^
AZO bottom/Cu +400 °C/AZO top	2.3 • 10^3^
AZO bottom/Cu +500 °C/AZO top	1.9 • 10^3^
Wet samples	Sheet resistance [Ω/Sq]
AZO bottom/Cu NP acetone/AZO top	2.5 • 10^6^
AZO bottom/Cu NP IPA/AZO top	4.2 • 10^4^

## Data Availability

All data concerning this work are reported in the present paper.
